# The Etiological Bacterial Spectrum of Neck Abscesses of Lymph Node Origin - Gram-Positive and Gram-Negative Bacteria

**DOI:** 10.7759/cureus.46940

**Published:** 2023-10-13

**Authors:** Yanko G Yankov

**Affiliations:** 1 Department of General and Operative Surgery, Medical University "Prof. Dr. Paraskev Stoyanov", Varna, BGR

**Keywords:** lymphogenic abscess, etiological spectrum, lymphadenopathy, maxillofacial surgery, oral surgery, neck, lymph node, purulent inflammation, bacteria, abscess

## Abstract

According to our medical practice as maxillofacial and oral surgeons, operated patients with purulent infections of the cervical lymph nodes are not many in number. On the other hand, the presence of a purulent infection requires not only the surgical evacuation of the pus but also the application of antimicrobial preparations. This necessitates good knowledge of the spectrum of the bacterial causative agents of the disease, the determination and analysis of which is the purpose of this original article. The bacteria studied in 181 patients with a mean age of 26.25 years, ranging between 29 days and 82 years, who underwent surgery for suppurating cervical lymph nodes, were retrospectively analyzed over a period of eight years. No bacteria were found in 69 of them. In 83 (74.11%) of the remaining 112 studied patients, the isolated microorganisms were of the gram-positive spectrum - *Staphylococcus aureus* (n=34), gram-positive resident microflora represented by more than one bacterial species (n=21), *Staphylococcus hemolyticus* (n=10), *Staphylococcus epidermidis* (n=9) and beta-hemolytic streptococci (n=9). Gram-negative bacteria were 25.89% (n=29) - *Klebsiella pneumoniae* (n=8), *Bartonella henselae* (n=7), *Klebsiella oxytoca* (n=6), *Enterobacter cloacae* (n=5) and *Flavimonas oryzihabitans* (n=3). No anaerobic and fungal microorganisms were isolated. Therefore, antimicrobial therapy in these patients should be directed against both gram-positive and gram-negative bacteria, which in our study were represented in a ratio of approximately three to one in favor of gram-positive microorganisms. Otherwise, we create a prerequisite for the formation of phlegmon on the neck, which hides real chances for the lives of patients.

## Introduction

According to data from our medical practice, 5.71% of hospitalized patients with diseases in the domain of maxillofacial surgery have cervical lymph node pathology, for which they sought help from a maxillofacial or oral surgeon. Of these, 15.75% (a total of 0.86% of all hospitalized patients) had purulent inflammation of an affected lymph node, of a group of adjacent lymph nodes, or with an abscess of the soft tissues adjacent to the inflamed lymph node. The goal of surgical treatment in these patients is to evacuate the formed pus with subsequent lavage and drainage of the affected anatomical lodge [1]. The surgical treatment in them is always accompanied by antibacterial application [2]. To choose the right antibiotic therapy, it is necessary to know the etiological bacterial spectrum of this purulent disease, the study and analysis of which is the aim of the present article. It examines and analyzes the etiological bacterial causative agents of patients hospitalized and operated on by us with purulent inflammation of the neck lymph nodes over a period of eight years.

## Materials and methods

The present study is retrospective. From the beginning of 2015 to the end of 2022, 1143 patients with diseases of the lymph nodes were treated in the Clinic of Maxillofacial Surgery of the University Multispecialty Hospital for Active Treatment "St. Marina" in the city of Varna, Bulgaria. One hundred eighty-two of them had clinical, laboratory, and imaging data for purulent inflammation of one or more cervical lymph nodes. Clinically, during the examination by an internist, pediatrician, and oral or maxillofacial surgeon, the typical local signs of inflammation, such as swelling, soreness, redness, and increased local temperature in the affected part of the neck, were observed. Laboratory tests showed increased values of C-reactive protein (CRP), erythrocyte sedimentation rate (ESR), neutrophils, and leukocytes. In each patient, an imaging study (ultrasound, magnetic resonance imaging, or computed tomography with or without contrast material) was performed preoperatively, which confirmed the diagnosis of purulent inflammation of a cervical lymph node or bundle of cervical lymph nodes, which provided reasonable evidence for proceeding to surgery.

One of the patients had known oncohematological disease and clinical and imaging evidence of enlarged cervical lymph nodes fused in bundles. During the surgical intervention, material was taken for histological examination, which proved the already known diagnosis of non-Hodgkin's lymphoma, and this patient was excluded from the study.

In the remaining 181 patients with an average age of 26.25, ranging between 29 days and 82 years, there were no anamnestic and clinical data on known lymph node diseases (oncohematological, metastatic, inflammatory, congenital, or acquired immune deficiency), they were not subjected to tissue and organ transplants and did not take immunosuppressive medications. In all of them, during the surgical treatment aimed at evacuating the purulent exudate, a sample was taken with a sterile swab to conduct a microbiological examination in order to identify the bacterial causative agent and create an antibiogram. The samples were examined by microbiology colleagues from the Clinic of Microbiology of the same university hospital in the city of Varna, Bulgaria. Each sample was subjected to direct gram microscopy and then confirmed by a biochemical machine analyzer "VITEK" (bioMérieux, Marcy-l'Étoile, France).

## Results

**Figure 1:**
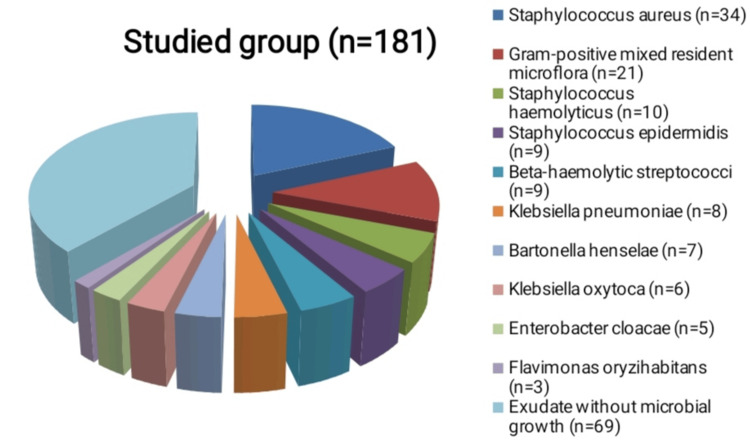
Schematic presentation of the isolated bacterial agents in the studied population of patients with purulent inflammation of the cervical lymph nodes

Of all 181 patients, no bacterial growth was isolated in 69, and the microbiological cultures remained sterile. Of the remaining 112, the largest part is the isolated causative agent *Staphylococcus aureus* (n=34), followed by representatives of the gram-positive mixed resident microflora (n=21). The following are other materials from which only one bacterial species was isolated - coagulase-negative staphylococci (n=19), of which *Staphylococcus haemolyticus* (n=10) and *Staphylococcus epidermidis* (n=9), beta-hemolytic streptococci (n=9 ), *Klebsiella pneumoniae* (n=8), *Bartonella henselae* (n=7), *Klebsiella oxytoca* (n=6), *Enterobacter cloacae* (n=5) *and Flavimonas oryzihabitans* (n=3) (Table 1, Figure 1).

**Table 1:** Distribution of the isolated bacteria in the patients with neck abscesses of lymph node origin

Number by order	Isolated bacteria	Gram positive (+) or negative (-)	Number of isolates (n)
1	Staphylococcus aureus	+	34
2	Gram-positive mixed resident microflora	+	21
3	Coagulase-negative staphylococci:	+	19
3.1	Staphylococcus haemolyticus	+	(10)
3.2	Staphylococcus epidermidis	+	(9)
4	Beta-haemolytic streptococci	+	9
5	Klebsiella pneumoniae	-	8
6	Bartonella henselae	-	7
7	Klebsiella oxytoca	-	6
8	Enterobacter cloacae	-	5
9	*Flavimonas* oryzihabitans	-	3
10	Exudate without microbial growth		69
	Total		181

## Discussion

With high virulence of the bacterial causative agent and/or weakened immune forces of the human organism and in the absence of antibacterial treatment, the inflammation of the affected lymph nodes passes from a serous to a suppurative stage. Initially, the capsule of the affected lymph node is intact, but subsequently, it ruptures, and pus flows from the interior of the lymph node into the surrounding soft tissue lodge. Inflammation of the lymph nodes goes through three successive stages. In the first, which is serious, the treatment is oral or parenteral administration of antibiotics. During the subsequent stages of inflammation of the lymph nodes, in which pus has already formed in the capsule of the lymph node (second stage), or its capsule has lysed and the pus has come out into the adjacent soft tissues (third stage), the therapy is not only antibacterial but also operative (evacuation of the available pus) [1,2]. It usually takes several days (usually two to five) for the final isolation of the pathogen causing the infection and the preparation of an antibiogram. During this time, the choice of antibiotic is made by the attending physician empirically. Therefore, in order to be able to correctly choose the antibacterial drug therapy for these patients, it is extremely important to know the etiological bacterial spectrum of the purulent inflammation of the cervical lymph nodes in humans.

According to our data, about one-third of all samples for microbiological examination do not isolate bacterial agents, so the number of sterile cultures in the patients we are discussing (n=69) is not unusual for us. Possible reasons for this are most likely improperly collected material by dipping the sterile swab into the core of the abscess focus, which is rich in pus but poor in bacteria, improper storage or transportation of the sample taken (at an inappropriate temperature, for example).

The typical causative agent of purulent inflammatory diseases, Staphylococcus aureus, was isolated in the largest number of examined patient samples (n=34), and this is not unusual.

Second in frequency is the gram-positive mixed bacterial resident microflora, which contains two or more different bacterial species (n=21), and this is typical of neck abscesses of any origin - odontogenic [3-5], rhinogenic [6], dermatogenic [2,6], posttraumatic [7].

Coagulase-negative staphylococci were isolated in a significant number of cultures (n=19). Their most common representatives causing human diseases are *Staphylococcus haemolyticus* and *Staphylococcus epidermidis* [8], which is also confirmed by our study, where no other representatives of this group were found. They are settled on the skin and mucous membranes of humans, where they do not cause infections under normal conditions [8]. However, with injuries to the skin and mucosal epithelium, as well as with immune disorders of the macroorganism, they can manifest themselves by causing purulent infections. They are less virulent than *Staphylococcus aureus*, which is also confirmed by our study [8,9], in which *Staphylococcus aureus* was detected almost twice as much as coagulase-negative staphylococci (34 versus 19).

Beta-hemolytic streptococci in humans colonize the distal parts of the gastrointestinal tract and do not cause disease under normal conditions [10,11]. They most often cause pharyngitis, especially in children [12,13]. They most likely get into the cervical lymph nodes and cause their purulent inflammation in respiratory tract infections, including asymptomatic ones.

Gram-negative bacteria are found significantly less often in neck infections, for which there is no specific reason [14]. Even in patients with carcinomas in this area, representatives from the gram-negative spectrum are single units only [15]. In our study, representatives of the gram-negative genus *Klebsiella *(n=14), of which *Klebsiella pneumoniae* (n=8) and *Klebsiella oxytoca* (n=6), were the most numerous - almost half of all isolated gram-negative microorganisms (14 of 29).

*Bartonella henselae* (n=7) causes cat scratch disease (CSD). In most cases, in the neck region in the adult individual, it manifests itself with serous inflamed lymph nodes, and rarely, it reaches the stage of purulent involvement [16]. More often, the formation of purulent exudate in the cervical lymph nodes is reached in children [17].

Enterobacter cloacae is a gram-negative normal inhabitant of the gastrointestinal tract in humans, and most likely, the purulent inflammation of the lymph nodes, in not a small number of the patients we describe (n=5), is due to an endogenous infection with this microorganism in patients with disorders of immunity [18]. There is evidence that it is a frequent cause of nosocomial infections, which could be another reason for its isolation in a relatively significant number of the studied by us patients [19,20].

*Flavimonas oryzihabitans* (n=3) is a gram-negative microorganism that most commonly causes infections in immunocompromised individuals [21,22] and nosocomial infections in patients with prolonged hospital stays [23]. There is literature data on its isolation in single patients with diabetes [24], tuberculosis [25], immunocompromised patients [24], and patients on chemotherapy [24]. It has been isolated from human wounds after animal bites, most commonly pigs [26], although the patients we describe lack anamnestic and clinical data of such animal incidents.

Although in our previous studies we found and described the isolation of anaerobic microorganisms in odontogenic and non-odontogenic head and neck abscesses [3-5], and although a number of other authors described the isolation of anaerobic bacteria in the microbiological analysis of suppurated lymph nodes [27-29], in our study of purulent inflammations of the lymph nodes of the neck, no such representatives were found. Fungi were also not isolated.

**Figure 2:**
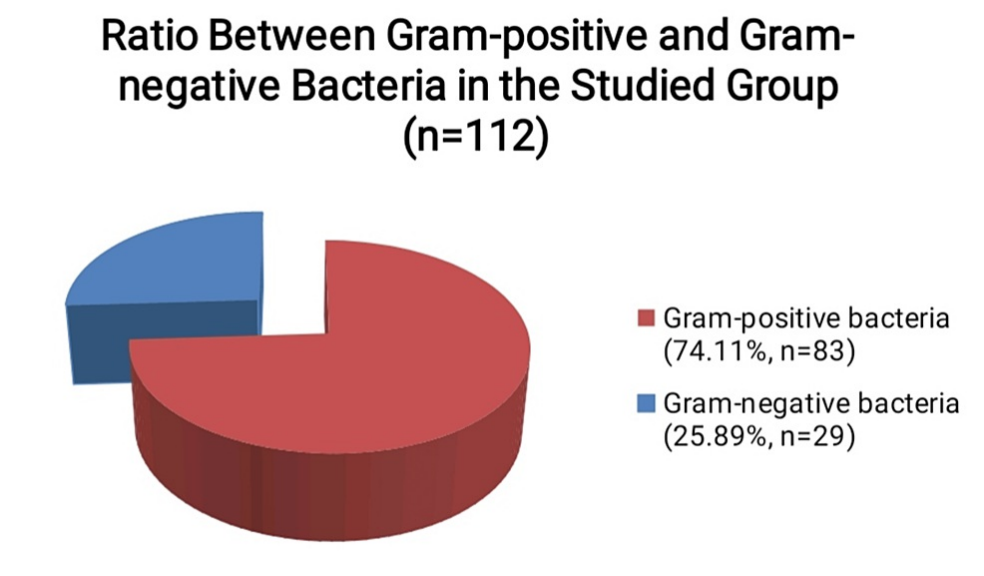
Percentage distribution of gram-positive and gram-negative bacteria isolated in the studied group of patients with purulent inflammation of the cervical lymph nodes, excluding the samples without microbial growth

The resident microflora isolated from the patients we describe is gram-positive. Most of the isolated causative agents are also gram-positive - *Staphylococcus aureus* (n=34), *Staphylococcus haemolyticus* (n=10), *Staphylococcus epidermidis* (n=9) and beta-hemolytic streptococci (n=9). Therefore, the total number of samples with isolated gram-positive microorganisms is 83 out of all 112 samples with isolates - 74.11%. Gram-negative microorganisms that were isolated from the studied patients were *Klebsiella pneumoniae* (n=8), *Bartonella henselae* (n=7), *Klebsiella oxytoca* (n=6), *Enterobacter cloacae* (n=5), and *Flavimonas oryzihabitans* (n=3). They are a total of 29, which is 25.89% (Figure 2).

Therefore, the antibacterial therapy that we empirically assign to patients with purulent cervical lymph node infections should be selected to cover the spectrum of both gram-positive and gram-negative bacteria, which in the present study is proved to occur in a ratio of approximately three to one in patients with this type of pathology.

This is the reason why we, as oral and maxillofacial surgeons in our clinical practice, prefer to prescribe antibacterial therapy that targets both gram-positive and gram-negative bacteria in patients with purulent neck lymph node infections. Otherwise, we run the risk that the purulent exudate can spread over the area and turn from an abscess into a phlegmon. The course of the disease in phlegmons in the maxillofacial area is usually severe, the treatment of these patients is long-term, and in many cases, it is carried out in intensive care units and clinics, and it is economically burdensome for the health system, and the prognosis is not always favorable, because the resulting complications in these patients carry a real risk of death.

## Conclusions

The main microorganisms causing purulent infections of the cervical lymph nodes in humans are gram-positive. Among them, the share of *Staphylococcus aureus *and gram-positive mixed resident microflora is the largest. Gram-positive bacteria are three times more numerous than gram-negative. The most common gram-negative representatives are of the genus *Klebsiella*, as well as the causative agent of cat-scratch disease *Bartonella henselae*. Therefore, antimicrobial therapy in these patients should be directed against both gram-positive and gram-negative microbacterial species.
